# Supplementary material for: Structured Populations of Critically Endangered Yellow Water Lily (Nuphar shimadai Hayata, Nymphaeaceae)
Source: Plants (Basel). 2022 Sep 19;11(18):2433. doi: 10.3390/plants11182433 (PMC9505410; doi:10.3390/plants11182433)

Table S1. The multilocus genotype (MLG) diversity of nipa populations in Indo-West Pacific[z].

Pop	N	MLG	eMLG	SE	H	G	lambda	E.5	Hexp	Ia	p.Ia	rbarD	p.rD
WP	22	22	11	4.45e-07	3.09	22	0.955	1	0.413	3.529	0.000999	0.11199	0.000999
GPb	13	13	11	0.00e+00	2.56	13	0.923	1	0.276	0.961	0.001998	0.04771	0.001998
GPa	11	11	11	0.00e+00	2.40	11	0.909	1	0.284	0.166	0.269730	0.00712	0.269730
GPn	16	16	11	0.00e+00	2.77	16	0.938	1	0.395	0.944	0.000999	0.02730	0.000999
Total	62	62	11	0.00e+00	4.13	62	0.984	1	0.416	1.646	0.000999	0.04518	0.000999

[z]Pop = population

N = census population size

MLG = number of unique multilocus genotype (MLG) observed

eMLG = number of expected MLG based on rarefaction at smallest N ≥ 10

SE = standard error of rarefaction analysis

H = Shannon-Wiener Index of MLG diversity

G = Stoddart and Taylor's Index of MLG diversity

lambda = Simpson's Index

E.5 = Evenness

Hexp = Nei's (1978) expected heterozygosity

Ia = Index of association

p.Ia = p-value for Ia

rbarD = standardized index of association

p,rD = p-value for rbarD

Table S2. Statistical analysis results of Mantel correlogram.

**Distance class (W)**	**Class index**	**Number of distances**	**Mantel correlation**	***p* values (Mantel)**	***p* values (corrected)**
1	41.451486	1254	0.444921	0.0010	0.0010***
2	124.354458	0	NA	NA	NA
3	207.257430	0	NA	NA	NA
4	290.160402	0	NA	NA	NA
5	373.063374	0	NA	NA	NA
6	455.966346	1056	-0.300587	0.0010	NA
7	538.869318	416	0.059146	0.2139	NA
8	621.772291	0	NA	NA	NA
9	704.675263	0	NA	NA	NA
10	787.578235	0	NA	NA	NA
11	870.481207	0	NA	NA	NA
12	953.384179	1056	-0.207566	0.0010	NA

Significance codes: *** 0.001

Table S3. The list of first-generation (F_0) immigrants detected among *N. shimadai* populations in the northern Taiwan based on multilocus genotypes[a].

**Sample**	**Home population**	**-log (L_home/L_max)**	**Probability**	**-log(L)**	**Reference population**
ns39	GPb	2.802	0.0006	11.759	GPa
ns41	GPa	1.498	0.0077	15.412	GPb
ns50	GPa	1.307	0.0037	22.204	GPb

[a] log(L-home/L_max) (likelihood ratio); probability, significant at p<0.01; -log(L) (genetic distance); reference population (population of origin) with population codes found in Table 5.

Table S4. Number of migrants (*Nm*) using private alleles.

**Sample size**	***Nm***
Mean N = 10	0.451128
Mean N = 25	0.269447
Mean N = 50	0.199791
Corrected value for sample size	0.291291

Mean sample size: 15.4872

Mean frequency of private alleles, p (1): 0.165214

Figure S1. PCR amplicons of the selected 10 primers

ACC-111

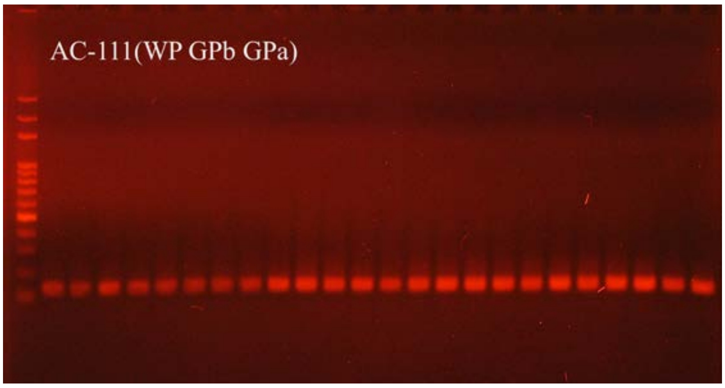

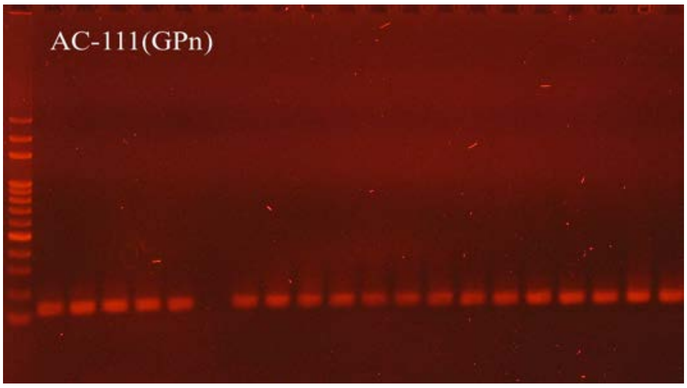

AC165

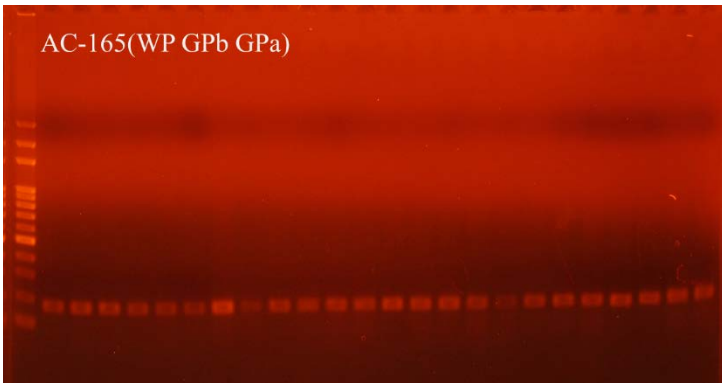

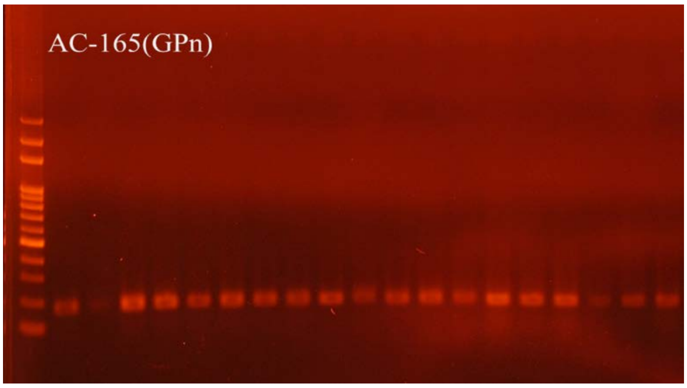

AC-176

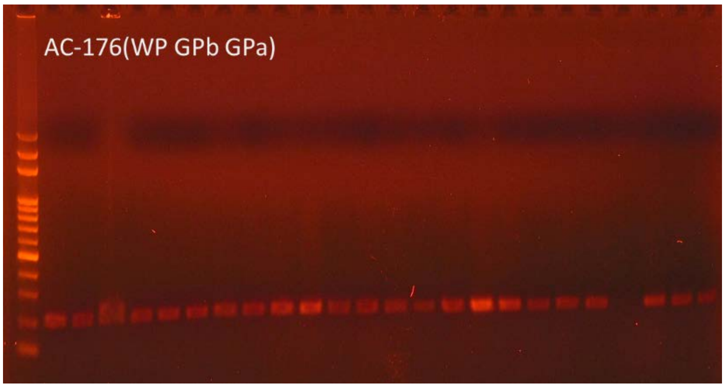

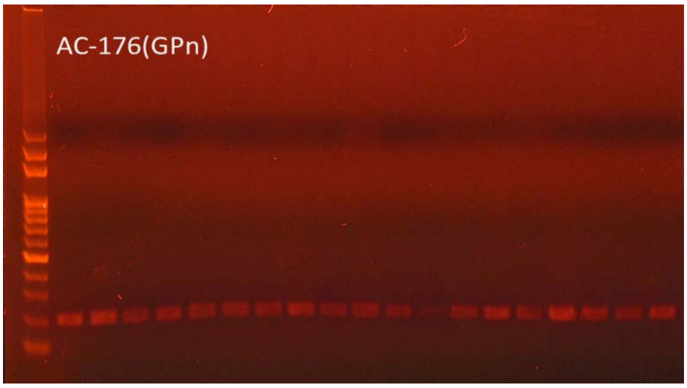

AC-178

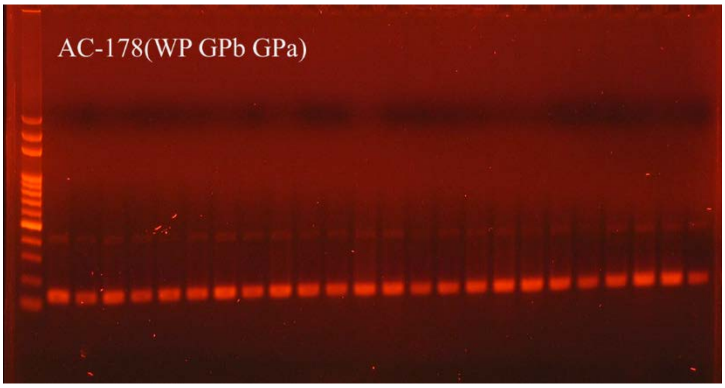

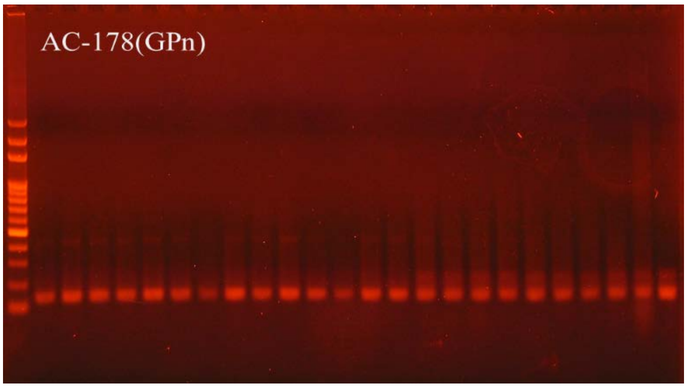

AC-182a

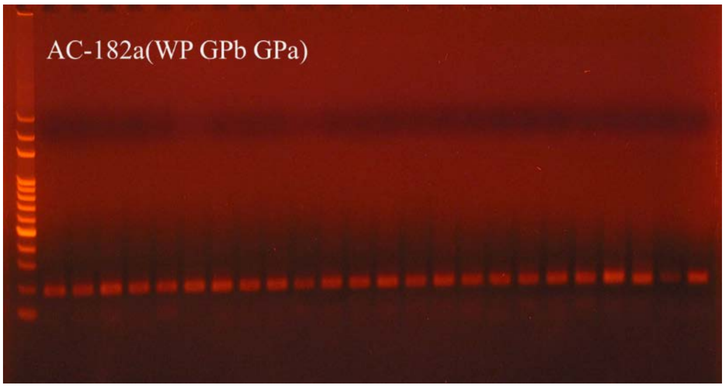

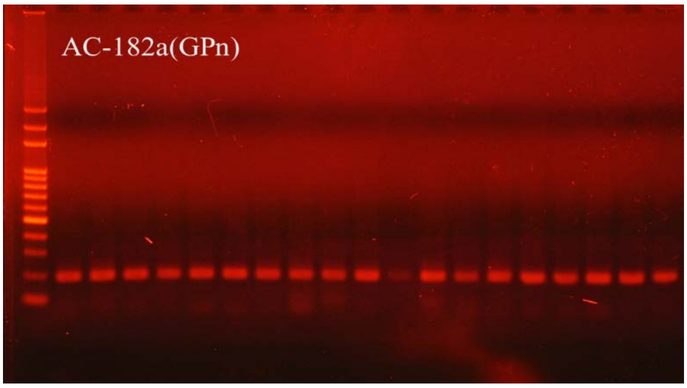

AC-182b

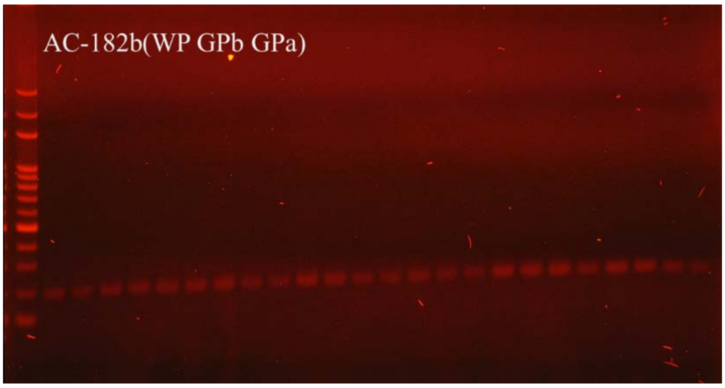

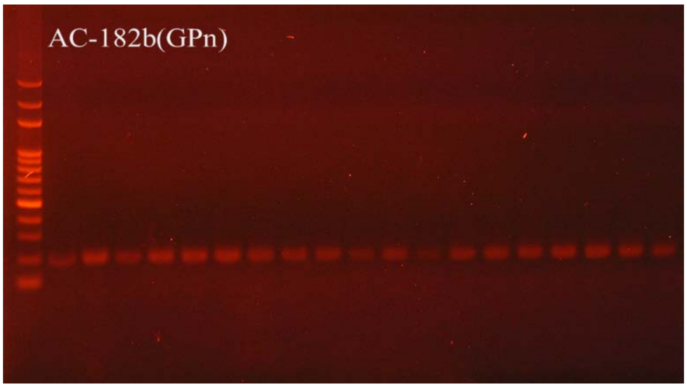

AC-186

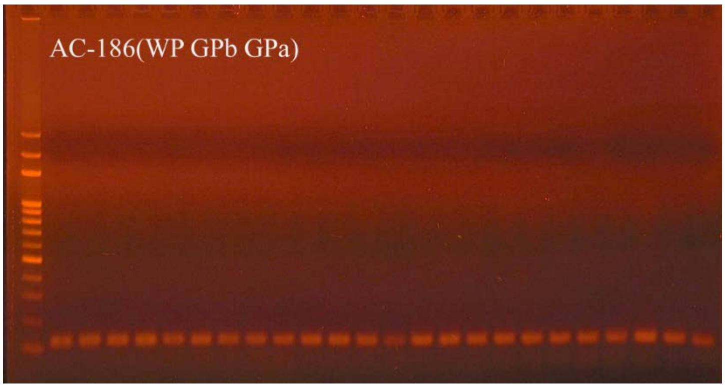

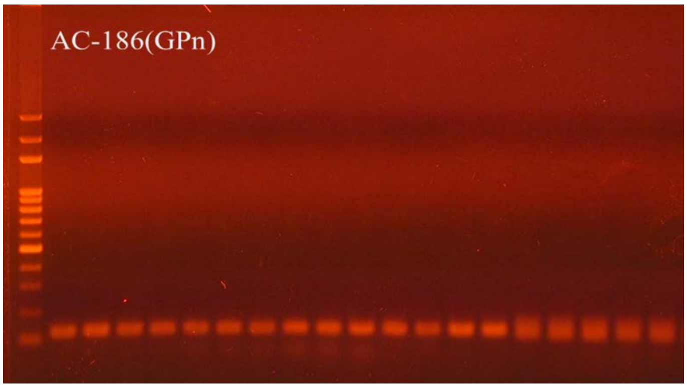

AG-135

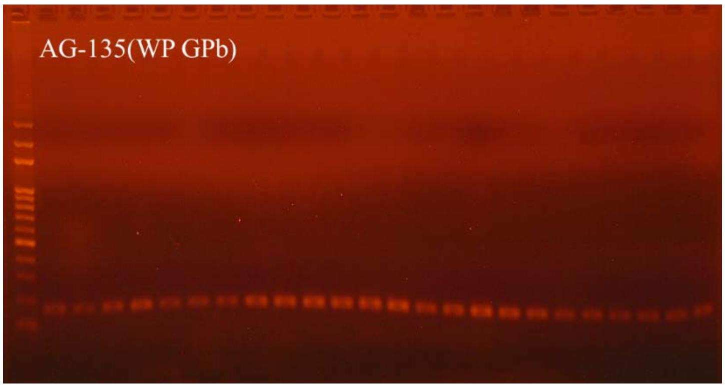

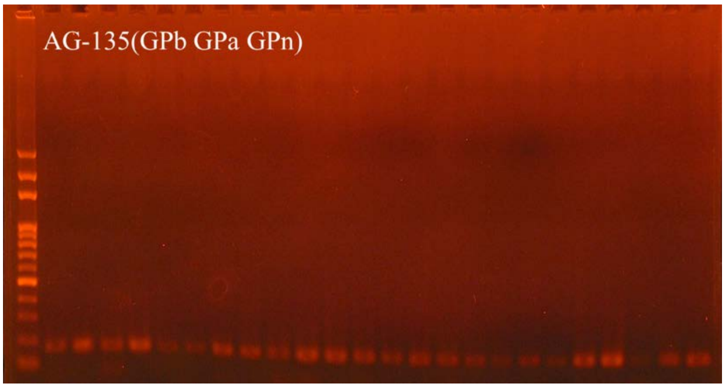

AG-223

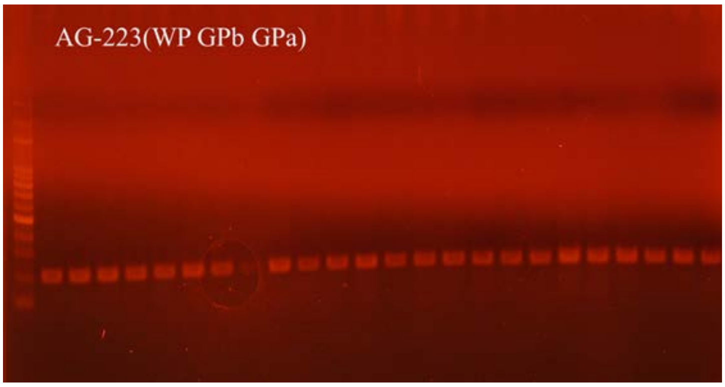

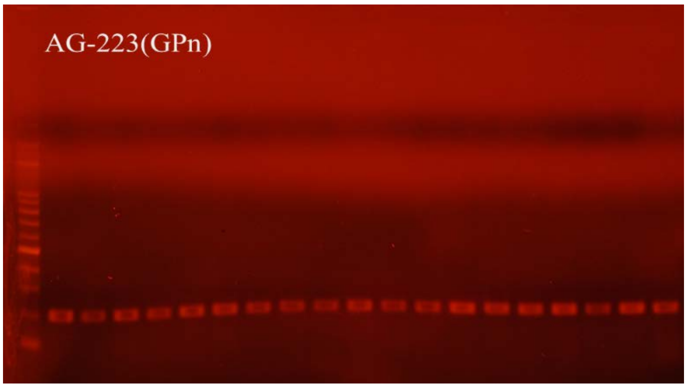

AG-224

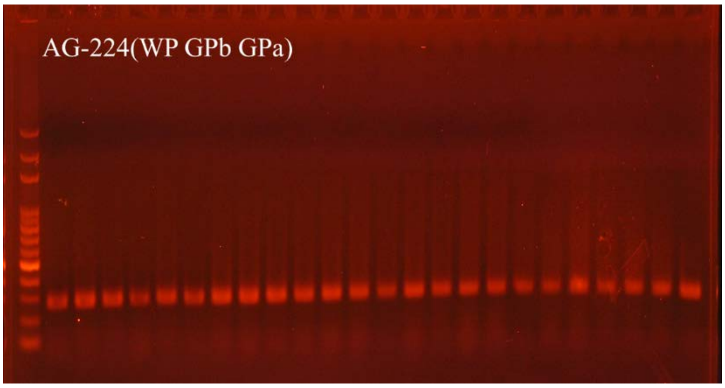

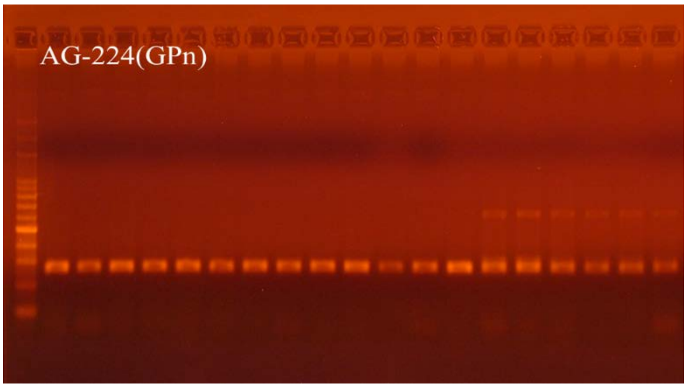

Supplement: Supplementary file 1 [file plants-11-02433-s001.zip › plants-1893830-supplementary.pdf]
